# Liquid biopsy for patients with IBD-associated neoplasia

**DOI:** 10.1186/s12885-020-07699-z

**Published:** 2020-12-03

**Authors:** Hideaki Kinugasa, Sakiko Hiraoka, Kazuhiro Nouso, Shumpei Yamamoto, Mami Hirai, Hiroyuki Terasawa, Eriko Yasutomi, Shohei Oka, Masayasu Ohmori, Yasushi Yamasaki, Toshihiro Inokuchi, Masahiro Takahara, Keita Harada, Takehiro Tanaka, Hiroyuki Okada

**Affiliations:** 1 Department of Gastroenterology and Hepatology, Okayama University Graduate School of Medicine, Dentistry, and Pharmaceutical Sciences, 2-5-1 Shikata-cho, Kita-ku, Okayama, 700-8558 Japan; 2 Department of Pathology, Okayama University Graduate School of Medicine, Dentistry, and Pharmaceutical Sciences, 2-5-1 Shikata-cho, Kita-ku, Okayama, 700-8558 Japan

**Keywords:** IBD-associated neoplasia, IBD-associated cancer, Liquid biopsy, ctDNA

## Abstract

**Background:**

It is often difficult to diagnose inflammatory bowel disease (IBD)-associated neoplasia endoscopically due to background inflammation. In addition, due to the absence of sensitive tumor biomarkers, countermeasures against IBD-associated neoplasia are crucial. The purpose of this study is to develop a new diagnostic method through the application of liquid biopsy.

**Methods:**

Ten patients with IBD-associated cancers and high-grade dysplasia (HGD) with preserved tumor tissue and blood were included. Tumor and non-tumor tissues were analyzed for 48 cancer-related genes using next-generation sequencing. Simultaneously, circulating tumor DNA (ctDNA) was analyzed for mutations in the target genes using digital PCR.

**Results:**

Out of 10 patients, seven had IBD-related cancer and three had IBD-related HGD. Two patients had carcinoma in situ; moreover, three had stageII and two had stage III. To avoid false positives, the mutation rate cutoff was set at 5% based on the control results; seven of 10 (70%) tumor tissue samples were mutation-positive. Mutation frequencies for each gene were as follows: TP53 (20.9%; R136H), TP53 (25.0%; C110W), TP53 (8.5%; H140Q), TP53 (31.1%; R150W), TP53 (12.8%; R141H), KRAS (40.0%; G12V), and PIK3CA (34.1%; R 88Q). The same mutations were detected in the blood of these seven patients. However, no mutations were detected in the blood of the remaining three patients with no tumor tissue mutations. The concordance rate between tumor tissue DNA and blood ctDNA was 100%.

**Conclusion:**

Blood liquid biopsy has the potential to be a new method for non-invasive diagnosis of IBD-associated neoplasia.

**Supplementary Information:**

The online version contains supplementary material available at 10.1186/s12885-020-07699-z.

## Background

It is widely accepted that cancers occur in patients suffering from IBD and these cancers are distinguished from sporadic colorectal cancers as IBD-associated cancers. Eaden et al. [1] reported the incidence rates of IBD-associated cancers as 2.1%/10 years, 8.5%/20 years, and 17.8%/30 years, while Rutter et al. [2] reported the incidence rates as 2.5%/20 years, 7.6%/30 years, and 10.8%/40 years. Although there is a slight variation in the rates of IBD-associated cancers from several reports [3], it is still a challenge to prevent IBD-associated cancers in patients suffering from IBD.

The functionality of colonoscopy as a surveillance method for the detection of IBD-associated cancers has been studied. Although its contribution to colorectal cancer detection has long been known, a systemic review revealed that colonoscopy for IBD diagnosis not only improves the detection rate of colorectal cancer but also its effectiveness in colorectal cancer-associated deaths [4]. However, endoscopic diagnosis of cancer in IBD is often difficult due to background inflammation, and countermeasures are imperative.

Randomized biopsy has been used as a surveillance method for IBD-associated cancers mainly in Europe and the United States, but the usefulness of targeted biopsy has also been reported with the development of endoscopic instruments [5]. In addition, the application of indigo staining and NBI (narrow band imaging) has been demonstrated in various studies and the surveillance methods have been reviewed [6, 7]. However, none of these methods provides a breakthrough for the existing methods and clinicians are still in search for the other alternatives and improvisations.

On the other hand, new findings about the genes involved in IBD-associated cancers have been reported in the last few years. Compared to the mutations involved in sporadic colorectal cancer, the involvement of APC mutations is lower and new findings such as the involvement of TP53, KRAS, SMAD4, and IDH1 have been revealed [8–12].

Liquid biopsy has been attracting attention as a non-invasive method for cancer treatment [13]. Traditionally, a direct biopsy of the tumor is considered to be the only way to diagnose cancer, but this new method of liquid biopsy is drawing attention because it can obtain tumor information from blood, which is comparable to that of the conventional methods [14]. Endoscopic diagnosis of cancer in IBD is often difficult due to the background inflammation and the lack of sensitive tumor biomarkers. The application of liquid biopsy appears to be the best for the detection of IBD-associated cancers.

The purpose of the current study was to examine and detect the presence of mutated genes in blood via circulating tumor DNA (ctDNA) of IBD-associated tumors and to examine the functionality of liquid biopsy by comparing tumor tissue DNA with ctDNA.

## Methods

### Patients and genes

Ten patients with IBD-associated cancers and high-grade dysplasia, treated at the Okayama University Hospital from 2004 to 2015, with preserved tumor tissue and blood were included in this study. Tumor tissues were analyzed using next-generation sequencers, while blood was simultaneously analyzed for mutations using digital PCR. We selected 48 cancer-related genes based on previously published information of whole genome analysis [10, 11]. These genes included the following: ABL1, AKT1, ALK, AR, ATM, BRAF, CDKN2A, CSF1R, CTNNB1, EGFR, ERBB2, ERBB4, FANCA, FANCC, FANCF, FANCG, FGFR1, FGFR2, FGFR3, FLT3, HRAS, IDH1, IDH2, JAK2, JAK3, KIT, KRAS, MAP 2 K1, MAP 2 K2, MAP 2 K4, MET, NOTCH1, NPM1, NRAS, PDGFRA, PIK3CA, PIK3R1, PTEN, RET, RUNX1, SMAD4, SMO, SRC, STK11, TP53, VHL, WT1, and hTERT. All patients provided written informed consent prior to enrolment. This study was approved by the ethics committee of Okayama University Hospital (1602–047/1506–070) and conducted in accordance with the Declaration of Helsinki.

Also, not only non-cancerous tissue samples paired to cancerous tissue samples but also ten patients with IBD (5 Crohn and 5 UC) without a tumor have been observed as control in this study.

### Extraction of DNA

Formalin fixed paraffin embedded (FFPE) samples were obtained by surgical resection. Histological examinations confirmed that these samples contained a minimum of 30% tumor cells. We extracted DNA from 5-μm thick sections from FFPE samples using a QIAamp DNA FFPE Tissue Kit (Qiagen, Valencia, CA, USA), according to the manufacturer’s instructions.

All blood samples were collected prior to initial treatment. DNA was extracted from each source according to the manufacturer’s instructions. Plasma samples were separated by centrifugation (3000 rpm, 10 min, 4 °C) within 3 h of blood collection. The samples were then stored at − 30 °C for subsequent DNA extraction. Cell free DNA (cfDNA) was extracted from 1 mL of plasma using the QIAamp Circulating Nucleic Acid Kit (Qiagen, Hilden, Germany). All DNAs were eluted in a final volume of 50 μL and stored at − 30 °C. DNAs extracted from plasma were quantified using a Qubit fluorometer (Thermo Fisher Scientific, Waltham, MA, USA).

### Next-generation sequencing (NGS)

We performed NGS (Miseq, illumina, Hayward, CA, USA) and deep targeted sequencing for 48 cancer-related genes in the target custom enrichment panel (Integrated DNA Technologies, Inc., Coralville, Iowa, USA). In the present study, geneious prime (Biomatters, Ltd., New Zealand) was used as the bioinformatics tools for the analyses. We set the cut-off value at 1% frequency. In addition to these cut-off values, we set the coverage over 1000. We excluded the genes for which mutations were detected in non-cancerous tissue samples in further analysis.

### Droplet digital PCR

The presence of mutations was detected via droplet digital PCR (QX200 system; Bio-Rad Laboratories, Hercules, CA, USA) using each probe that was designed from the results of NGS. The probes used are as follows: TP53 R136H, KIT N818H, BRAF I452S, ERBB2 V812I, ATM L3017R, STK11 L195M, PIK3CA R88Q, STK11 R333C, ERBB2 V812L, TP53 C110W, SMAD4 E330D, AR Y225S, MAP 2 K4 R134Q, PDGFRA F837V, PDGFRA I843S, KRAS G12V, KIT W553C, KIT Y819S, TP53 R150W, ATM L2890I, RUNX1 P403L, TP53 H140Q, MAP 2 K4 S184L, MAP 2 K4 V321M, TP53 R141H, RUNX1 N233T, IDH2 R172G, PDGRFA R558H, AR C612S, and FGFR3 K537E. DNA eluent (5 μL) from plasma was mixed with Droplet PCR Supermix (10 μL; Bio-Rad Laboratories, Hercules, CA, USA), primer/probe mixture (2 μL), and sterile DNase- and RNase-free water (5 μL). The mixture (22 μL) was added to droplet generation oil (70 μL; Bio-Rad Laboratories, Hercules, CA, USA) to produce a droplet. The emulsion was subjected to PCR in a thermal cycler and the conditions were as follows: an initial temperature of 95 °C for 10 min, 40 cycles at 94 °C for 30 s, and 55 °C for 1 min. A final step was performed at 98 °C for 10 min for enzyme deactivation and the reaction mixtures were analyzed using a droplet reader (Bio-Rad Laboratories, Hercules, CA, USA). For quantification, fluorescence signal readout was obtained using QuantaSoft software (Bio-Rad Laboratories, Hercules, CA, USA). In this study, we set the minimum number of events used to call positivity at one droplet, and the threshold of fluorescence intensity at 1200 using a positive control and a negative control, which were artificial gene synthesis products. All experiments were conducted in duplicates to diminish the effect of a false negative result, although its probability was low in these materials. A result was considered positive even if it occurred once.

## Results

### Patient characteristics

There were eight cases of ulcerative colitis and two cases of Crohn’s disease. The median age of the patients was 49 years, comprising of four males and six females. Out of the 10 patients, seven had IBD-associated cancer and three had high-grade dysplasia. Two patients had carcinoma in situ; moreover, three had stageII and two had stageIII (Table 1).

**Table 1:** Patient characteristics

	Patient					Neoplasia					Values of biomarkers		
Case no.	IBD type	Age (years)	Sex	Extent of disease	Duration (years)	Location	Type	Histology	Stage	Treatment	CEA (ng/mL)	CA19-9 (U/mL)	CRP (mg/dL)
1	CD	42	M	ileocolonic	15	Rectum	5	Muc	3	Surgery	3.7	10.5	7.3
2	CD	70	M	Ileal	20	Ileum	5	Por	2	Surgery	1.9	27.4	0
3	UC	35	M	Pancolitis	26	Ascending	2	Por	2	Surgery	2.7	10.8	0.05
4	UC	62	F	Pancolitis	36	Sigmoid	5	Muc	3	Surgery	3.0	18.2	0.2
5	UC	48	F	Pancolitis	25	Rectum	5	Muc	2	Surgery	1.2	8.8	3.7
6	UC	40	F	Pancolitis	14	Rectum	0-IIb	HGD	–	Surgery	1.7	30.9	0.02
7	UC	49	F	Left-sided	7	Sigmoid	0-IIa + IIb	HGD	–	ESD	1.3	9.4	0.04
8	UC	83	F	Pancolitis	17	Rectum	0-IIa	HGD	–	Surgery	1.5	7.6	0.4
9	UC	53	M	Pancolitis	30	Rectum	0-IIc	Tub1	Tis	Surgery	2.7	8.1	0.05
10	UC	34	F	Left-sided	14	Rectum	0-Ia	Pap	Tis	Surgery	0.2	10.5	0.06

*Abbreviation*: *IBD* inflammatory bowel disease, *UC* Ulcerative colitis, *CD* Crohn disease, *M* Male, *F* Female, *I* Ileum, *A* ascending colon, *S* sigmoid colon, *R* Rectum, *ESD* Endoscopic submucosal dissection, *tub1* well differentiated tubular adenocarcinoma, *tub2* moderately differentiated tubular adenocarcinoma, *pap* papillary adenocarcinoma, *muc* mucinous adenocarcinoma, *por* poorly differentiated adenocarcinoma, *HGD* high grade dysplasia, *Tis* carcinoma in situ

The patient background for each of the cases is as follows: cases 1 and 2 have Crohn’s disease; cases 3 to 10 have ulcerative colitis; cases 1 to 5 have advanced cancer; cases 6 to 10 have carcinoma in situ and/or HGD. Tumor DNA mutation analysis was performed on these cases using next-generation sequencers (Table 1).

### The NGS analysis

The NGS analysis of tumor tissue DNA showed the following results: in case 1, TP53 was 20.9%; in case 2, PIC3CA was 34.1%; in case 3, TP53 was 25.7%; in case 4, KRAS was 40%; in case 5, TP53 was 31.1%; in case 6, TP53 was 8.5%; in case 7, TP53 was 12.8%; in case 8, IDH2 was 1.1%; in case 9, AR was 1.5%; and in case 10, FGFR3 was 1.0% in frequency (Table 2).

**Table 2:** The next**-**generation sequencing and the digital PCR analysis in case 1 to 10

Case no.	Tumor tissue DNA						Blood ctDNA					
**1**												
Gene	**TP53**	KIT	BRAF	ERBB2	ATM	STK11	**TP53**	KIT	BRAF	ERBB2	ATM	STK11
AA	**R136H**	N818H	I452S	V812I	L3017R	L195M	**R136H**	–	–	–	–	–
Freq(%)	**20.9**	1.7	1	1.2	1.2	1.2	**3.1**	–	–	–	–	–
**2**												
Gene	**PIK3CA**	STK11	ERBB2				**PIK3CA**	STK11	ERBB2			
AA	**R88Q**	R333C	V812L				**R88Q**	–	–			
Freq(%)	**34.1**	2.6	2.4				**5.3**	–	–			
**3**												
Gene	**TP53**	SMAD4	AR	MAP 2 K4	PDGFRA	PDGFRA	**TP53**	SMAD4	AR	MAP 2 K4	PDGFRA	PDGFRA
AA	**C110W**	E330D	Y225S	R134Q	F837V	I843S	**C110W**	–	–	–	–	–
Freq(%)	**25.7**	2.2	1.8	1.4	1	1	**7.8**	–	–	–	–	–
**4**												
Gene	**KRAS**	KIT	KIT				**KRAS**	KIT	KIT			
AA	**G12V**	W553C	Y819S				**G12V**	–	–			
Freq(%)	**40**	1.2	1.1				**7.7**	–	–			
**5**												
Gene	**TP53**	ATM	RUNX1				**TP53**	ATM	RUNX1			
AA	**R150W**	L2890I	P403L				**R150W**	–	–			
Freq(%)	**31.1**	3.3	1.1				**50.7**	–	–			
**6**												
Gene	**TP53**	MAP 2 K4	MAP 2 K4				**TP53**	MAP 2 K4	MAP 2 K4			
AA	**H140Q**	S184L	V321M				**H140Q**	–	–			
Freq(%)	**8.5**	2.1	1.1				**3.9**	–	–			
**7**												
Gene	**TP53**	RUNX1					**TP53**	RUNX1				
AA	**R141H**	N233T					**R141H**	–				
Freq(%)	**12.8**	9.1					**6.3**	–				
**8**												
Gene	IDH2	PDGFRA					IDH2	PDGFRA				
AA	R172G	R558H					–	–				
Freq(%)	1.1	1					–	–				
**9**												
Gene	AR						AR					
AA	C612S						–					
Freq(%)	1.5						–					
**10**												
Gene	FGFR3						FGFR3					
AA	K537E						–					
Freq(%)	1						–					

*Abbreviation*: *AA* amino acid, *Freq* frequency, *ctDNA* circulating tumor DNA

The NGS of control showed that there were no genes more than 3% in frequency (Supplemental 1).

### The digital PCR analysis

The following are the results of digital PCR analysis of blood ctDNA: detection of TP53 in case 1, PIC3CA in case 2, TP53 in case 3, KRAS in case 4, TP53 in case 5, TP53 in case 6, and TP53 in case 7. However, we were unable to detect cases 8, 9, and 10, which had low mutation frequencies in tumor tissue DNA. Interestingly, the high frequency of the tumor tissue DNA mutation, especially those with a frequency of 5% or higher, could be detected by blood ctDNA. Amino acid substitutions due to mutations in tissue DNA and blood ctDNA also correlated with each other, thus suggesting that the ctDNA in these cases originated in tumor tissue DNA (Table 2).

If we consider more than 5% of the tumor tissue DNA mutations to be positive, it would result in three wild type and seven mutation cases. For these cases, the results of blood ctDNA detection via liquid biopsy also confirmed three wild type and seven mutation cases. Therefore, the concordance rate was 100% (Table 3).

**Table 3:** Comparison between tumor tissue DNA and blood ctDNA

	Blood ctDNA	
Tumor tissue DNA	Wild	Mutation (> 1%)
Wild	3	0
Mutation (> 5%)	0	7

Liquid biopsy in ten patients with ulcerative colitis without a tumor showed there was no detected genes (Supplemental 1).

## Discussion

In this study, the detection of blood ctDNA in IBD-associated tumors was performed. The concordance rate between tumor tissue DNA and blood ctDNA was 100%, thereby suggesting the possibility of a non-invasive method. Interestingly, liquid biopsy was also useful for high-grade dysplasia cases, but a certain frequency of tumor tissue DNA mutations was required for the detection of blood ctDNA mutations.

Our previous studies revealed that ctDNA mutations require more than 10% frequency rate of target tumor tissue DNA mutations [15–17]. The current results, therefore, corroborated the previous studies. Although cancerous lesions and non-cancerous lesions in patients with IBD had a few types of mutations due to carcinogenesis and inflammation, the key driver mutations had much higher frequency. For this reason, liquid biopsy might be an ideal method because only high frequency mutations could be detected by blood ctDNA.

Although the utility of liquid biopsy has recently been demonstrated in various fields, there have been no reports on detection of IBD-associated neoplasia using this technique. This may be due to the lack of centers that handle large numbers of cases and the need to target a large number of genes. However, this is the first study to report the use of this technique for the detection of IBD-associated neoplasia.

There are few limitations in this study. First, this is a single-center, small-scale, retrospective, observational study, so that a multicenter, large-scale, prospective study will be required. Based on these results, a prospective validation study is underway. Second, the study evaluates only 48 genes and higher detection rate can be achieved by combining other genetic mutations in future studies. Third, the origin of gene mutation in blood is unknown. This hinders the clinical application of liquid biopsy; however, in this study, we compared tumor tissue DNA and blood ctDNA mutations and even confirmed a match between the mutation locations, and therefore, conclude that the ctDNA was derived from cancer tissues. Forth, although DNA is usually stable for long time, we have not examined the effect of the storage time on this analysis. The storage period might be one of the limitations in future studies. Fifth, cfDNA quantity in blood might limit the detection in early stage disease. Sixth, although biopsy samples were not used in this study, laser microdissection might help to capture the dysplastic areas in that case.

## Conclusion

Blood liquid biopsy has the potential to be a new method for non-invasive diagnosis of IBD-associated tumors and may develop as one of the next-generation detection techniques in future. Although further molecular biological analysis will be required to narrow down the candidate genes for future use in screening, it could be a very useful method for monitoring.

## Supplementary Information

**Additional file 1: Supplemental 1.** The next-generation sequencing and the digital PCR analysis in control 1 to 10 (Crohn: 1–5; UC: 6–10).

## Data Availability

The datasets used and analysed during the current study are available from the corresponding author on reasonable request.

## Abbreviations

IBD: Inflammatory bowel disease
HGD: High-grade dysplasia
ctDNA: Circulating tumor DNA
NBI: Narrow band imaging
FFPE: Formalin fixed paraffin embedded
cfDNA: Cell free DNA
NGS: Next-generation sequencing
