# Supplementary material for: Liquid biopsy for patients with IBD-associated neoplasia
Source: BMC Cancer. 2020 Dec 3;20:1188. doi: 10.1186/s12885-020-07699-z (PMC7712625; doi:10.1186/s12885-020-07699-z)
Supplement: Supplementary file 1 — Additional file 1: Supplemental 1. The next-generation sequencing and the digital PCR analysis in control 1 to 10 (Crohn: 1–5; UC: 6–10). [file 12885_2020_7699_MOESM1_ESM.docx]

**Supplemental 1. The next-generation sequencing and the digital PCR analysis in control 1 to 10 (Crohn: 1-5; UC: 6-10).**

| **Control  no.** |  |  |  | **Tissue DNA** | | | | | |  | **Blood cfDNA** | | | | | |
| --- | --- | --- | --- | --- | --- | --- | --- | --- | --- | --- | --- | --- | --- | --- | --- | --- |
| **1** |  | Gene |  | PIK3R1 | RET | TP53 | SKT11 | RUNX1 |  |  | PIK3R1 | RET | TP53 | SKT11 | RUNX1 |  |
|  |  | AA |  | T213M | C618F | E11D | R333H | E395K |  |  | - | - | - | - | - |  |
|  |  | Freq(%) |  | 1.2 | 1.2 | 1.2 | 1.2 | 1.3 |  |  | - | - | - | - | - |  |
|  |  | Gene |  | FANCG |  |  |  |  |  |  | FANCG |  |  |  |  |  |
| **2** |  | AA |  | V471G |  |  |  |  |  |  | - |  |  |  |  |  |
|  |  | Freq(%) |  | 1.1 |  |  |  |  |  |  | - |  |  |  |  |  |
|  |  | Gene |  | TP53 | AR | WT1 |  |  |  |  | TP53 | AR | WT1 |  |  |  |
| **3** |  | AA |  | R158H | H158N | H240Y |  |  |  |  | - | - | - |  |  |  |
|  |  | Freq(%) |  | 1.9 | 1.1 | 1.4 |  |  |  |  | - | - | - |  |  |  |
|  |  | Gene |  | RUNX1 |  |  |  |  |  |  | RUNX1 |  |  |  |  |  |
| **4** |  | AA |  | E395K |  |  |  |  |  |  | - |  |  |  |  |  |
|  |  | Freq(%) |  | 1.0 |  |  |  |  |  |  | - |  |  |  |  |  |
|  |  | Gene |  | SKT11 |  |  |  |  |  |  | SKT11 | ATM | RUNX1 |  |  |  |
| **5** |  | AA |  | G346S |  |  |  |  |  |  | - | - | - |  |  |  |
|  |  | Freq(%) |  | 1.0 |  |  |  |  |  |  | - | - | - |  |  |  |
|  |  | Gene |  | WT1 |  |  |  |  |  |  | WT1 |  |  |  |  |  |
| **6** |  | AA |  | H240Y |  |  |  |  |  |  | - |  |  |  |  |  |
|  |  | Freq(%) |  | 1.2 |  |  |  |  |  |  | - |  |  |  |  |  |
|  |  | Gene |  | CTNNB1 | PIK3R1 | BRAF | HRAS | MAP 2K4 | RUNX1 |  | CTNNB1 | PIK3R1 | BRAF | HRAS | MAP 2K4 | RUNX1 |
| **7** |  | AA |  | S29Y | A313T | T485K | A11T | T318I | E429K |  | - | - | - | - | - | - |
|  |  | Freq(%) |  | 1.0 | 2.6 | 1.2 | 1.1 | 1.2 | 2.0 |  | - | - | - | - | - | - |
|  |  | Gene |  | EGFR | WT1 |  |  |  |  |  | EGFR | WT1 |  |  |  |  |
| **8** |  | AA |  | T785A | Q142K |  |  |  |  |  | - | - |  |  |  |  |
|  |  | Freq(%) |  | 1.0 | 1.1 |  |  |  |  |  | - | - |  |  |  |  |
|  |  | Gene |  | EGFR | BRAF | SKT11 |  |  |  |  | EGFR | BRAF | SKT11 |  |  |  |
| **9** |  | AA |  | V786L | T458K | G276D |  |  |  |  | - | - | - |  |  |  |
|  |  | Freq(%) |  | 1.4 | 1.2 | 1.7 |  |  |  |  | - | - | - |  |  |  |
|  |  | Gene |  | PTEN | WT1 | MAP 2K4 | RUNX1 |  |  |  | PTEN | WT1 | MAP 2K4 | RUNX1 |  |  |
| **10** |  | AA |  | C304R | Q238H | P338L | T410A |  |  |  | - | - | - | - |  |  |
|  |  | Freq(%) |  | 1.5 | 1.0 | 1.8 | 1.1 |  |  |  | - | - | - | - |  |  |

Abbreviation: AA, amino acid; Freq, frequency; cfDNA, cell free DNA
